# *Helicobacter pylori* infection promotes the invasion and metastasis of gastric cancer through increasing the expression of matrix metalloproteinase-1 and matrix metalloproteinase-10

**DOI:** 10.3892/etm.2014.1822

**Published:** 2014-07-03

**Authors:** HAO JIANG, YUJUAN ZHOU, QIANJIN LIAO, HONGJUAN OUYANG

**Affiliations:** 1 Gastric Cancer Research Center of Hunan, Department of Oncology, The First Affiliated Hospital, University of South China, Hengyang, Hunan 421001, P.R. China; 2 Hunan Cancer Hospital and the Affiliated Cancer Hospital of Xiangya School of Medicine, Central South University, Changsha, Hunan 410013, P.R. China

**Keywords:** gastric cancer, *Helicobacter pylori*, matrix metalloproteinase-1, matrix metalloproteinase-10, metastasis

## Abstract

The aim of this study was to investigate the effect and mechanism of *Helicobacter pylori* infection in the invasion and metastasis of gastric cancer. Specimens from 80 patients with gastric cancer (of which 20 patients had metastatic gastric cancer) and 40 patients with chronic gastritis were included in this study. *H. pylori* infection was determined by ELISA and the expression of matrix metalloproteinase-1 (MMP-1) and MMP-10 was observed using immunohistochemistry. The correlation between *H. pylori* infection and the clinical pathological features of gastric cancer was analyzed by SPSS 13.0 software. The protein expression levels of MMP-1 and MMP-10 in MGC-803 cells infected with *H. pylori* were analyzed using western blotting. *H. pylori* infection was found in 62 of the 80 patients with gastric cancer and in 13 of the 40 patients with chronic gastritis. In addition, *H. pylori* infection was correlated with the staging and lymph node metastasis, but not with the gender, age and histological types of patients. *H. pylori* infection was also significantly correlated with the expression of MMP-1 and MMP-10 (r=0.8718, P<0.05 and r=0.5477, P<0.05, respectively). The expression of MMP-1 and MMP-10 was significantly upregulated following induction by *H. pylori* infection (P<0.05), with significant effects occurring following infection for 12 and 6 h, respectively. *H. pylori* infection may promote the invasion and metastasis of gastric cancer by increasing the expression of MMP-1 and MMP-10.

## Introduction

The development of gastric cancer involves several factors, among which *Helicobacter pylori* infection is the most important (1). *H. pylori* is a type of micro-aerobic, Gram-negative, spiral bacterium, which resides between the gastric mucous layer and the gastric surface. *H. pylori* secretes urease, which damages the gastric mucosal barrier. The *H. pylori* lipopolysaccharide inhibits the binding of laminin to its receptor, resulting in gastric mucosal injury (2). The vacuolating toxin gene of *H. pylori* can change ion permeability, leading to cell degeneration (3), and damage the gastric mucosa, causing erosion or ulceration. *H. pylori* expresses cytotoxin-associated gene (Cag) A, which generates a cytotoxic effect and induces inflammatory and immune responses. The deformation and necrosis of mucosal cells and inflammatory infiltration can be observed in *H. pylori*-infected lesions, and specific antibodies can be detected in serum (4,5).

Matrix metalloproteinases (MMPs) are highly homologous, zinc-dependent endopeptidases that can degrade the basement membrane. To date, 19 types of MMPs have been identified. All MMPs are secreted by mesenchymal cells in the form of a protease precursor, and can be inhibited by tissue inhibitors of metalloproteinases (TIMPs). It has been found that MMPs are important in tumor invasion, metastasis, cardiovascular disease and diabetes, and are closely associated with the degradation of the extracellular matrix (ECM) of tumor cells (6).

Invasion and metastasis are the prominent features of malignant tumors, and the degradation of the ECM is one of the key steps involved in these processes. Matrix degradation primarily depends on proteolytic enzymes. An increasing number of studies have shown that the invasion and metastasis of tumor cells are closely associated with MMP production. *In vitro* and *in vivo* studies have shown that MMPs are functional in various stages of tumor progression, influencing tumor genesis, growth, angiogenesis and metastasis (7–9). The interaction of MMPs with the basement membrane is the initiating signal of tumor invasion and metastasis.

As mentioned above, tumor invasion and metastasis involve the secretion of MMPs. MMP-1 predominantly degrades the stroma, which is closely associated with local invasion and metastasis (10,11). MMP-10 is considered to be the most important factor in human tumor cells that can activate other MMP (such as MMP-1) precursors (12). MMP-10 can also degrade certain interstitial proteins, contributing to metastasis (13).

In this study, in order to determine the effect of *H. pylori* infection on gastric cancer, the associations among *H. pylori* infection, the expression of MMP-1 and MMP-10 and the invasion and metastasis of gastric cancer were investigated. *H. pylori* infection and MMP-1 and MMP-10 expression were detected in gastric cancer and chronic gastritis specimens. The association between *H. pylori* infection and the clinical pathological features of gastric cancer were then analyzed. Additionally, the expression of MMP-1 and MMP-10 following *H. pylori* infection in MGC-803 cells was studied.

## Materials and methods

### Cell line and reagents

The MGC-803 human gastric cancer cell line, a poorly differentiated gastric adenocarcinoma, was provided by the Department of School of Biological Science, Shandong Normal University (Jinan, China) and preserved in the Central Laboratory of the First Affiliated Hospital of Nanhua University (Hengyang, China). MGC-803 cells were cultured in high-glucose Dulbecco’s modified Eagle’s medium (DMEM) supplemented with 10% fetal bovine serum, 100 U/ml penicillin and 100 mg/ml streptomycin at 37°C in a humidified incubator with 5% CO_2_.

A Streptavidin-peroxidase (SP) immunohistochemistry kit, hematoxylin stain and 3,3′ diaminobenzidine (DAB) chromogenic agent were all purchased from Fuzhou Maixin Biotechnology Development Co., Ltd. (Fuzhou, China). Mouse anti-human MMP-1 and MMP-10 monoclonal antibodies for immunohistochemistry and western blotting were purchased from Santa Cruz Biotechnology, Inc. (Santa Cruz, CA, USA). Anti-mouse immunoglobulin G (IgG) was purchased from Boster Biological Technology, Ltd. (Wuhan, China). The ELISA kit was obtained from Bio-Check, Inc. (Foster City, CA, USA) and the bicinchoninic acid assay reagent was purchased from Pierce Chemical Co. (Rockford, IL, USA).

### Patient samples

Between 2005 and 2009, 80 cases of surgically resected and pathologically diagnosed gastric cancer paraffin specimens from the First Affiliated Hospital of Nanhua University were collected. The patients with gastric cancer were aged between 23 and 78 years, with a mean age of 53.2±12.7 years, and included 58 males and 22 females. Prior written and informed consent was obtained from every patient and the study was approved by the Ethics Review Board of Nanhua University. Of the 80 cases of gastric cancer, there were 20 cases of highly differentiated carcinoma, 20 cases of moderately differentiated carcinoma and 40 cases of poorly differentiated carcinoma. A total of 20 cases of gastric cancer exhibited lymph node metastasis. With regard to the extent of infiltration, there were 15 cases of early gastric cancer with gastric mucosal tissue infiltration limited to the mucosa or submucosal layer (regardless of lymph node metastasis) and 65 cases of advanced gastric cancer. Forty specimens of chronic superficial gastritis were collected as the control group. All specimens were fixed in formalin and embedded in paraffin. Specimens were sliced into 5-μm sections.

### Infection of MGC-803 cells with H. pylori

A total of 1×10^5^ MGC-803 cells were seeded in T75 tissue culture flask and 6 ml serum-free DMEM was added. After 24 h of synchronization, the culture medium was discarded. *H. pylori* culture medium was then added. According to the method described in a previous study (4), bacteria density was adjusted to 1×10^8^ CFU/ml, and two-fold diluted with serum-free DMEM. The MGC-803 cells were then co-cultured with *H. pylori* for 6, 12, 24 and 48 h.

### ELISA

Fasting blood (2 ml) was collected from all patients. For *H. pylori* IgG detection, ELISA was performed according to the manufacturer’s instructions (Bio-Check, Inc.). *H. pylori* IgG levels >20 U/ml were considered to be a positive result.

### Immunohistochemistry

Immunohistochemical staining (the SP method) was conducted to detect the protein expression of MMP-1 and MMP-10 in gastric cancer and chronic gastritis samples. Briefly, the samples were fixed in formaldehyde and embedded in paraffin. The sections were dewaxed, rehydrated in graded alcohols and processed prior to incubation with antibodies. Subsequent to blocking, the sections were incubated with primary antibodies at 37°C in the dark for 1 h. Secondary antibodies were then added and incubated in dark for 30 min following washing with phosphate-buffered saline (PBS). The sections were then developed with DAB chromogenic reagent and counterstained with hematoxylin. The working concentration of MMP-1 and MMP-10 primary antibody was 1:200. PBS was used in the negative control group instead of primary antibody. Positive staining for MMP-1 and MMP-10 was located in the cytoplasm. A total of ≥10 high-power fields were randomly selected (magnification, ×200), and ≥1,000 cells were counted. The staining intensity and percentage of positive cells was scored in each slice. Staining intensity was defined as 0 points for no staining, 1 point for pale yellow, 2 points for brownish yellow and 3 points for tan. The positive staining cell ratio was defined as 0 points for no staining, 1 point for <30%, 2 points for 30–60% and 3 points for >60%. The combined points total for the staining intensity and positive staining ratio was furthermore defined as follows: 0–2 points, weak positive or negative; 3–4 points, positive; and 5–6 points, strong positive. The rate of positive staining was calculated using the following formula: Positive rate = (weak positive case number + strong positive case number)/total cases × 100%.

### Western blotting

At 6, 12, 24 and 48 h after *H. pylori* infection, total proteins were extracted from MGC-803 cells and separated using SDS-PAGE. The proteins were then transferred onto a nitrocellulose membrane. Subsequent to blocking with non-fat milk, the membrane was incubated with monoclonal mouse anti-MMP-1 and -MMP-10 primary antibodies overnight at 4°C. The membrane was then washed and incubated with the anti-mouse IgG secondary antibody at 37°C for 1 h, prior to being developed using enhanced chemiluminescence plus reagent (Amersham, GE Healthcare Life Sciences, Piscataway, NJ, USA). β-actin was used as an internal control.

### Statistical analysis

SPSS 13.0 (SPSS, Inc., Chicago, IL, USA) software was used for statistical analysis. The immunohistochemical results were compared using a χ^2^ test. The Spearman’s rho correlation test was used to evaluate the correlations between *H. pylori* infection and MMP-1 and MMP-10 expression in gastric cancer. P<0.05 was considered statistically significant.

## Results

### MMP-1 and MMP-10 protein expression in gastric cancer is higher than that in chronic gastritis

**Figure 1:**
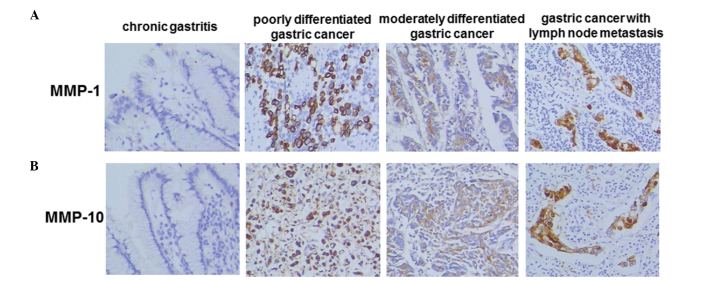
Immunohistochemical analysis of (A) MMP-1 and (B) MMP-10 protein expression in gastric cancer and chronic gastritis tissue. The expression of MMP-1 and MMP-10 was detected by immunohistochemical staining in tissues of chronic gastritis, poorly and moderately differentiated gastric cancer and gastric cancer with lymph node metastasis. Representative immunohistochemical staining images are shown (magnification, ×200). MMP, matrix metalloproteinase.

To determine MMP-1 and MMP-10 protein expression in gastric cancer and chronic gastritis, immunohistochemical staining was performed. Representative immunohistochemical staining results are shown in Fig. 1 and quantitative results are shown in Table I. As shown in Fig. 1, MMP-1 and MMP-10 expression was not detected in the chronic gastritis specimens. However, positive staining for MMP-1 and MMP-10 was detected in the poorly and moderately differentiated gastric cancer and gastric cancer with lymph node metastasis specimens. The positive staining rate was calculated as described in the Materials and methods section. As shown in Table I, the MMP-1-positive rate in patients with gastric cancer, metastatic gastric cancer and chronic gastritis was 78.8% (63/80), 95.0% (19/20) and 25.0% (10/40), respectively. The MMP-10-positive rate in the patients with gastric cancer, metastatic gastric cancer and chronic gastritis was 83.4% (67/80), 95.0% (19/20) and 30.0% (12/40), respectively. Statistically, the expression levels of MMP-1 and MMP-10 were significantly higher in the gastric cancer specimens than those in the chronic gastritis specimens (P<0.05). Furthermore, compared with levels in the gastric cancer specimens, the expression levels of MMP-1 and MMP-10 in the metastatic gastric cancer specimens were significantly higher (P<0.05). Therefore, these results show that expression levels of MMP-1 and MMP-10 were significantly increased in gastric cancer and particularly in metastatic gastric cancer.

### H. pylori infection rate in gastric cancer is higher than that in chronic gastritis

To detect the *H. pylori* infection level in gastric cancer and chronic gastritis specimens, fasting blood was collected and *H. pylori* IgG levels were measured by ELISA. There were 62 cases of *H. pylori* infection out of 80 gastric cancer cases with a positive rate of 77.5%, and 13 cases of *H. pylori* infection out of 40 chronic gastritis cases with a positive rate of 32.5%. Significantly higher levels of *H. pylori* infection were observed in the gastric cancer specimens than those in the chronic gastritis specimens (P<0.05, data not shown). This result suggests that there was significant *H. pylori* infection in gastric cancer.

### Correlation between H. pylori infection, MMP-1 and MMP-10 protein expression and gastric cancer pathological characteristics

To determine the roles of *H. pylori* infection and MMP-1 and MMP-10 protein expression in gastric cancer, the association between these factors and the clinical pathological characteristics of gastric cancer were analyzed. As shown in Table II, positive *H. pylori* infection and positive expression of MMP-1 and MMP-10 were associated with lymph node metastasis and clinical stage (P<0.05), but not with age, gender or differentiation degree (P>0.05). To further analyze the associations among *H. pylori* infection and MMP-1 and MMP-10 expression, correlation analyses were performed. It was revealed that MMP-1 and MMP-10 expression was significantly positively correlated in gastric cancer (r=0.8321, P<0.05; data not shown). *H. pylori* infection and MMP-1 and MMP-10 expression were also significantly positively correlated (r=0.8718, P<0.05 and r=0.5477, P<0.05, respectively; data not shown). Thus, these data suggest that associations exist among *H. pylori* infection, MMP-1 expression and MMP-10 expression in the development and metastasis of gastric cancer.

### MMP-1 and MMP-10 expression in H. pylori-infected gastric cancer cells is upregulated

**Figure 2:**
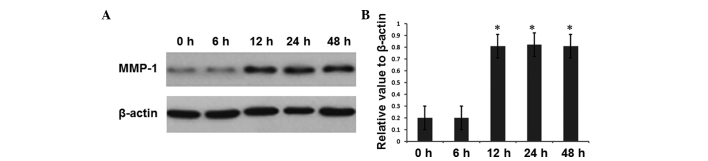
MMP-1 protein expression following *Helicobacter pylori* infection in MGC-803 gastric cancer cells. MMP-1 expression was detected by western blotting at 0, 6, 12, 24 and 48 h after *H. pylori* infection. (A) Representative western blotting results. (B) Quantitative western blotting results. ^*^P<0.05 compared with the value at 0 h. MMP-1, matrix metalloproteinase-1.

**Figure 3:**
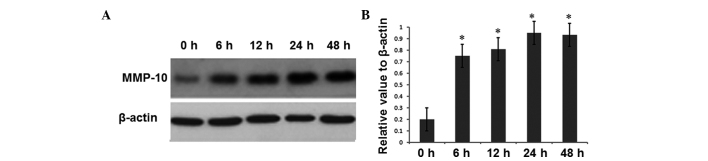
MMP-10 protein expression following *Helicobacter pylori* infection in MGC-803 gastric cancer cells. MMP-10 expression was detected by western blotting at 0, 6, 12, 24 and 48 h after *H. pylori* infection. (A) Representative western blotting results. (B) Quantitative western blotting results. ^*^P<0.05 compared with the value at 0 h. MMP-10, matrix metalloproteinase-10.

To investigate the effect of *H. pylori* infection on MMP-1 and MMP-10 expression, the expression levels of MMP-1 and MMP-10 were detected in MGC-803 cells following *H. pylori* infection. Western blotting was conducted at 6, 12, 24 and 48 h of infection. The results for MMP-1 and MMP-10 are shown in Figs. 2 and 3, respectively. As shown in Fig. 2, at 6 h of *H. pylori* infection, the intracellular MMP-1 expression level was not significantly changed, while at 12 h of *H. pylori* infection, the intracellular MMP-1 expression was significantly increased compared with that in the uninfected group at 0 h (P<0.05). MMP-1 expression at 24 and 48 h was maintained at a similar level to that at 12 h, which was also significantly increased compared with expression in the uninfected group at 0 h (P<0.05). However, the intracellular MMP-10 expression was significantly increased at 6 h of *H. pylori* infection compared with that in the uninfected group at 0 h (P<0.05) (Fig. 3). Similarly, MMP-10 expression at 12, 24 and 48 h of *H. pylori* infection remained significantly increased (P<0.05). These results indicate that *H. pylori* infection promoted the expression of MMP-1 and MMP-10 in gastric cancer cells.

## Discussion

MMP-1 is predominantly involved in the degradation of stromal components (10), and is important in tumor invasion and metastasis (11). MMP-10 is considered an important factor in the activation of other MMP (such as MMP-1) precursors in human tumor cells (12). In addition, MMP-10 can degrade proteins, including collagen III/IV/V, gelatin, nidogen, laminin-l and proteoglycans, thereby contributing to metastasis (13). This study has shown that MMP-1 and MMP-10 expression was higher in patients with gastric cancer with metastasis than in those with gastric cancer without metastasis. MMP-1 and MMP-10 expression in patients with gastric cancer and in those with metastatic gastric cancer was significantly higher than that in patients with gastritis. The positive expression of MMP-1 and MMP-10 was associated with lymph node metastasis and clinical stage, but not with age, gender or differentiation degree, indicating that MMP-1 and MMP-10 expression was involved in gastric cancer metastasis. It was also found that MMP-1 expression was correlated with MMP-10 expression, indicating that these two different types of MMPs may play a synergic role in gastric cancer genesis, invasion and metastasis. Thus, the simultaneous detection of MMP-1 and MMP-10 may be important in the evaluation of the prognosis and metastasis of gastric cancer.

Crawford *et al* (14) revealed that MMP-7 levels were significantly increased in *H. pylori*-Cag-positive gastric cancer cells, while not significantly increased in uninfected cells. Gööz *et al* (15) found that *H. pylori* can stimulate the secretion of MMP-1, MMP-3 and TIMP-3 in gastric cancer cells *in vitro*. However, TIMP-2 expression levels were not significantly changed. In the present study, it was revealed that *H. pylori* infection was significantly associated with MMP-1 and MMP-10 expression. Positive *H. pylori* infection was also associated with lymph node metastasis and clinical stage, and the number of specimens positive for *H. pylori* infection in advanced gastric cancer was significantly higher than that in early stage disease. This may be a result of the destruction of the normal mucosal barrier following *H. pylori* infection, stimulating MMP-1 and MMP-10 expression. MMPs can destroy the matrix-degrading balance, promote cancer invasion through the histological barrier (constituted by the basement membrane and ECM) into the surrounding tissues and metastasis to distant tissues, causing gastric cancer metastasis.

The present data showed that co-culturing *H. pylori* with MGC-803 cells led to the secretion of MMP-1 and MMP-10 by the MGC-803 cells, indicating that *H. pylori* infection may be involved in metastasis through the upregulation of MMP-1 and MMP-10 expression. At 6 h after *H. pylori* infection, MMP-10 expression was upregulated, while at 12 h MMP-1 began to be expressed. MMP-1 and MMP-10 expression was maintained at a high level at 24 and 48 h after *H. pylori* infection. As shown in previous studies (12,16), MMP-10 can activate precursors of other MMPs (including MMP-1). In this study, the results indicate that in the *H. pylori*-induced MMP secretion in gastric cancer cells, MMP-10 may be involved in the activation of MMP-1.

In conclusion, *H. pylori* infection is not only the primary factor of gastric cancer that causes mucosal injury, promotes tumor suppressor gene mutation and activates oncogenes, but may also be involved in invasion and metastasis by promoting MMP secretion, leading to gastric cancer deterioration and a decrease in survival time. *H. pylori* infection may be involved in gastric cancer metastasis through the mechanism of upregulating the expression of MMP-1 and MMP-10.

## Figures and Tables

**Table I:** MMP-1 and MMP-10 expression in specimens of gastric cancer and chronic gastritis.

Type of MMP and specimen	Cases (n)	Immunohistochemical score			P-value

		Weak positive, 0–2 (n)	Positive, 3–4 (n)	Strong positive, 5–6 (n)	
MMP-1					
Gastric cancer	80	17	19	44	0.004^a^
Metastatic gastric cancer	20	1	1	18	<0.001^b^
Chronic gastritis	40	30	9	1	<0.001^c^
MMP-10					
Gastric cancer	80	13	19	48	0.036 a
Metastatic gastric cancer	20	1	2	17	<0.001^b^
Chronic gastritis	40	28	10	2	<0.001^c^

Values of P<0.05 were determined using a one-way analysis of variance least significant difference test:

a Gastric cancer versus metastatic gastric cancer;

b metastatic gastric cancer versus chronic gastritis and

c gastric cancer versus chronic gastritis.

MMP, matrix metalloproteinase.

**Table II:** Correlation analysis of *Helicobacter pylori* infection, MMP-1 and MMP-10 protein expression and gastric cancer pathological characteristics.

		*H. pylori* infection			MMP-1 expression			MMP-10 expression		
							
Parameter	Cases (n)	Positive (n)	Negative (n)	P-value	Positive (n)	Negative (n)	P-value	Positive (n)	Negative (n)	P-value
Age in years				0.217			0.432	67	13	0.157
≥50	61	49	12		48	13		53	8	
<50	19	13	6		15	4		14	5	
Gender				0.597			0.532			0.098
Male	58	45	13		46	12		51	7	
Female	22	17	5		17	5		16	6	
Lymph node metastasis				<0.001^a^			<0.001^a^			<0.001^a^
With	52	48	4		48	4		50	2	
Without	28	14	14		15	13		17	11	
Clinical stage				<0.001^a^			<0.001^a^			<0.001^a^
Early	15	4	11		5	10		7	8	
Middle	65	58	7		58	7		60	5	
Differentiation degree				0.395			0.500			0.500
Poorly	40	32	8		31	9		33	7	
Highly and moderately	40	30	10		32	8		34	6	

a P<0.05, determined using a χ^2^ test.

MMP, matrix metalloproteinase.
